# Supplementary material for: Mapping the Photochemistry of European Mid-Latitudes Rivers: An Assessment of Their Ability to Photodegrade Contaminants
Source: Molecules. 2020 Jan 20;25(2):424. doi: 10.3390/molecules25020424 (PMC7024250; doi:10.3390/molecules25020424)
Supplement: Supplementary file 1 [file molecules-25-00424-s001.pdf]

SUPPLEMENTARY MATERIAL

Mapping the photochemistry of European mid-latitudes rivers: An assessment of their ability to photodegrade emerging contaminants

Luca Carena [1] and Davide Vione [1,*]

1 Dipartimento di Chimica, Università di Torino, Via Pietro Giuria 5, 10125 Torino, Italy.

* Correspondence: *davide.vione@unito.it*; Tel.: +39-011-6705296

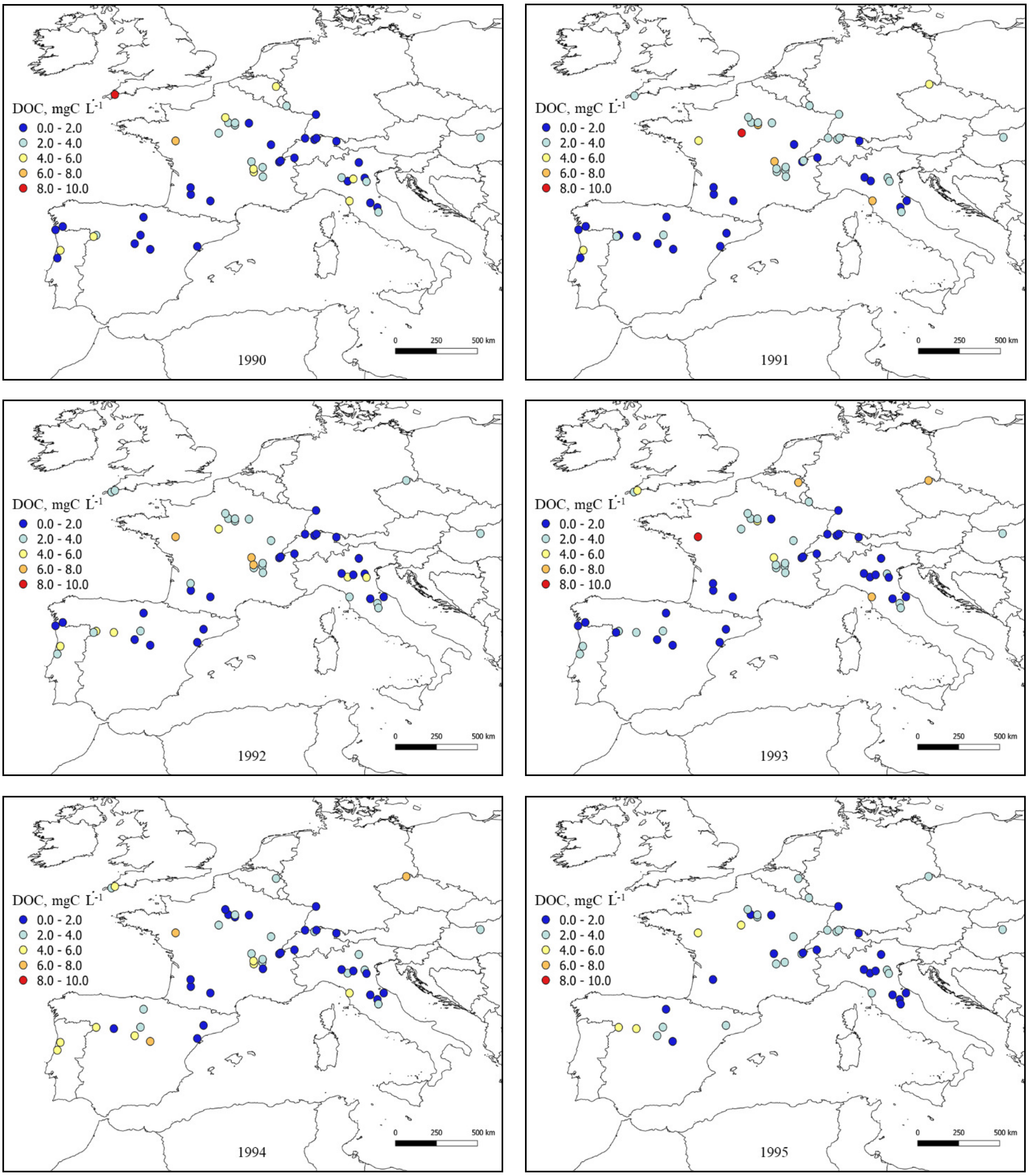

Fig. S1. DOC maps for European rivers belonging to the latitude belt from 40 to 50°N. Data are referred to the month of June and were from GEMStat (Global Environment Monitoring System). Maps were made by means of the QGIS software (version 3.2.2 'Bonn').

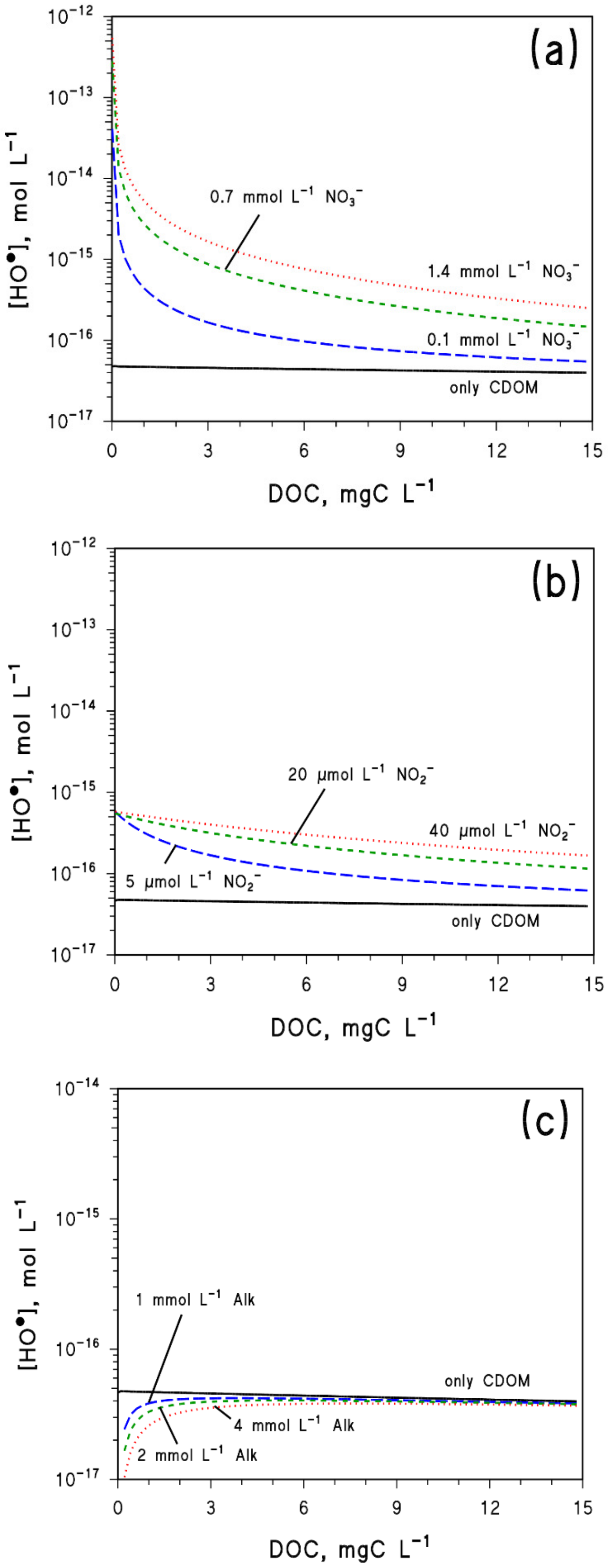

Fig. S2. Modeling the effects on the steady-state HO• concentrations of the addition of **(a)** nitrate, **(b)** nitrite and **(c)** bicarbonate/carbonate anions to a CDOM solution (black line). Data are referred to the month of June. Water optical depth = 0.1 m.

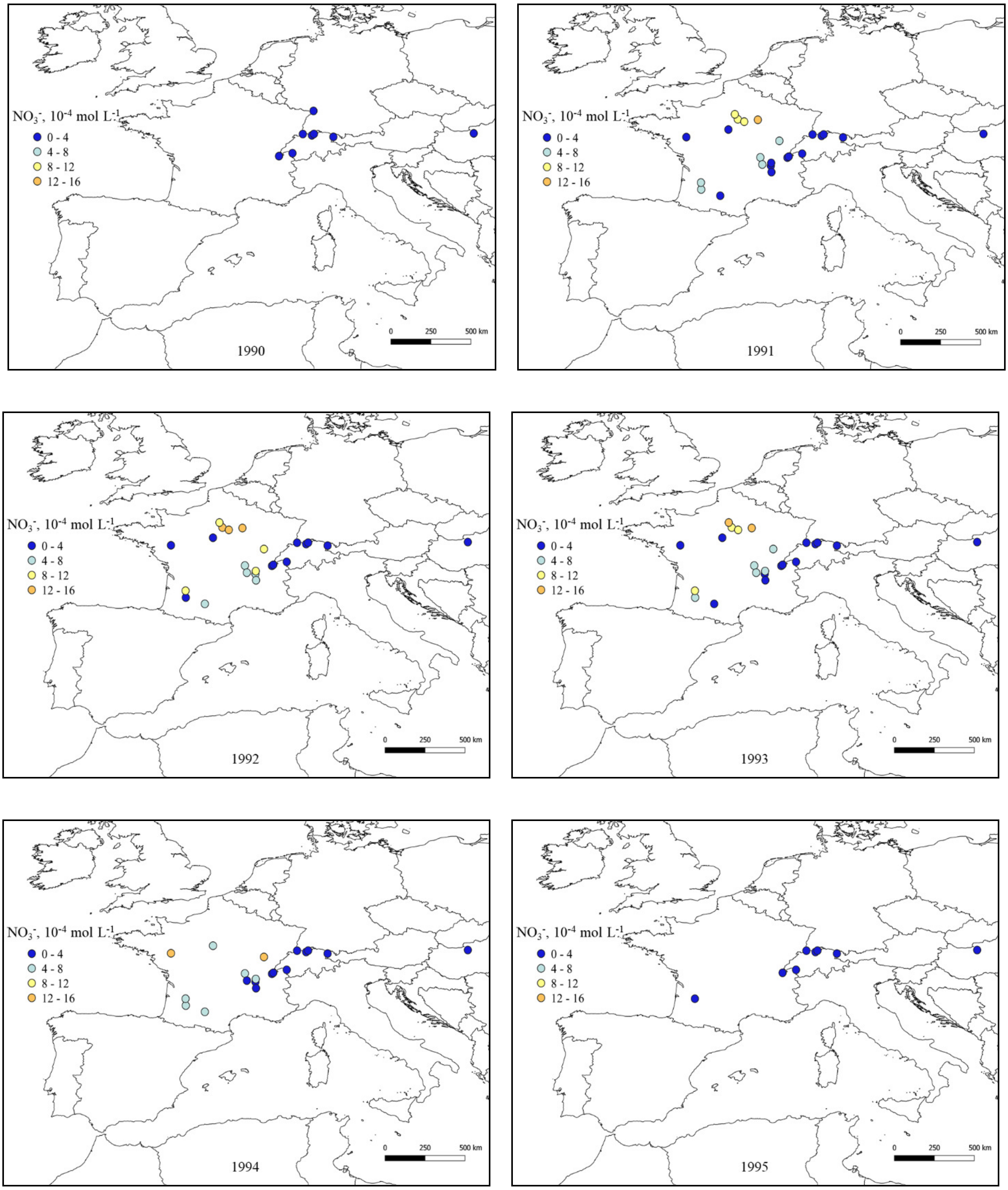

Fig. S3. Nitrate maps for European rivers belonging to the latitude belt from 40 to 50°N. Data are referred to the month of June and were from GEMStat (Global Environment Monitoring System). Maps were made by means of the QGIS software (version 3.2.2 'Bonn').

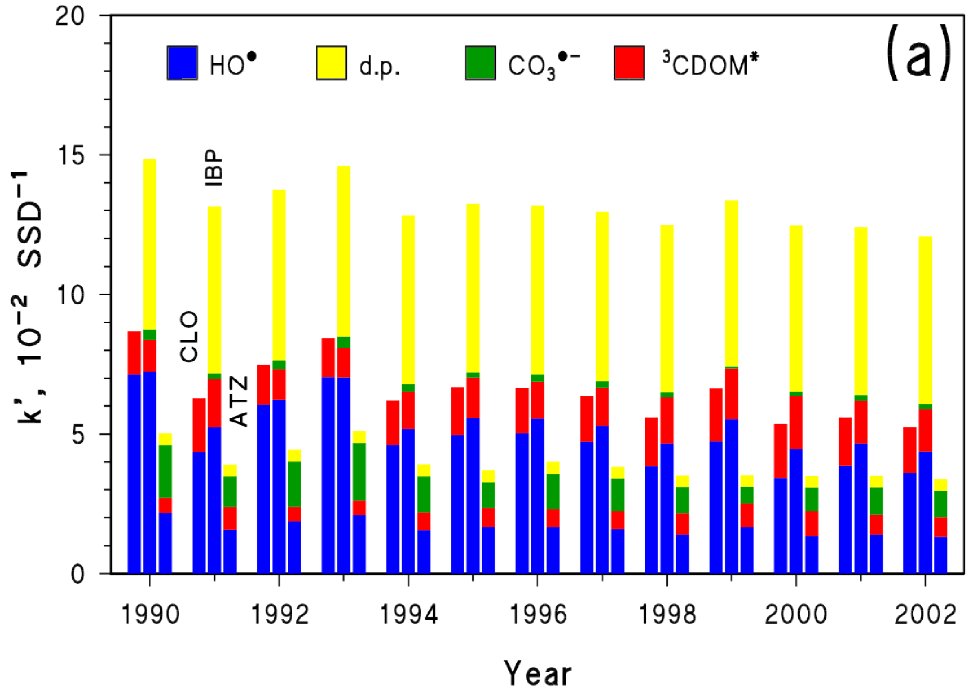
(a)
HO•
d.p.
CO₃•−
³CDOM*
CLO
IBP
ATZ
k', 10⁻² SSD⁻¹
Year

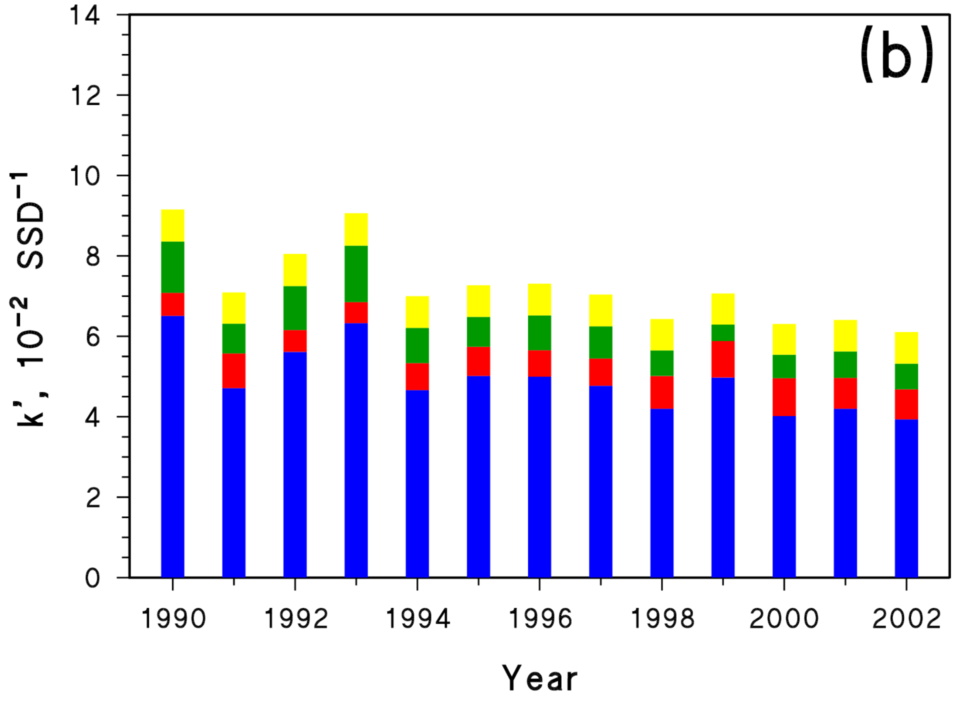
(b)
k', 10⁻² SSD⁻¹
Year

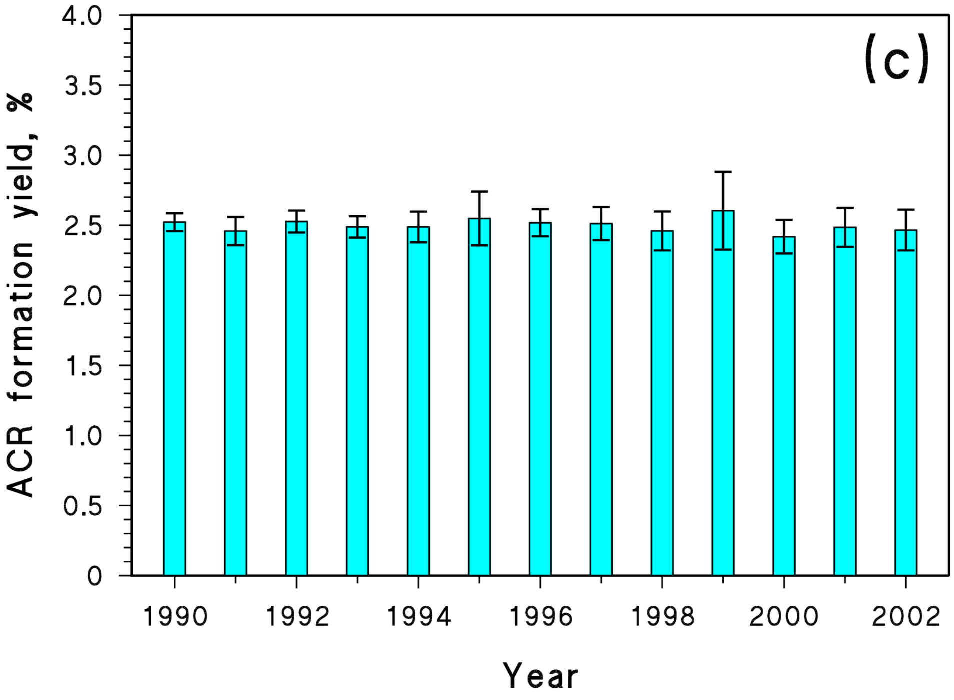

Fig. S4. Time trends of the photochemical pseudo-first order degradation rate constants (k') for **(a)** CLO, IBP, ATZ and **(b)** CBZ in Swiss rivers (0.1 m as optical water depth), during the month of June 1990-2002 (SSD = sunny summer days). Light blue bars in (c) are the formation yields (%) of acridine (ACR) from CBZ direct photolysis and HO• radicals reactions. They were calculated as the ratio $k_f\,(k')^{-1} = (\eta_{dp}\, k'_{dp} + \eta_{HO^\bullet}\, k_{HO^\bullet+CBZ}\, [HO^\bullet])(k')^{-1}$, where $\eta_{dp} = 0.036$ and $\eta_{HO^\bullet} = 0.031$ are the formation yields of ACR by CBZ direct photolysis and HO• radicals reactions, respectively (De Laurentiis et al., 2012). Note that k_f is the formation rate constant of ACR, while k′ is the pseudo-first order degradation rate constant of CBZ accounted for by both direct photolysis and indirect photochemistry ($k' = k'_{dp} + k_{HO^\bullet+CBZ}\, [HO^\bullet]$).

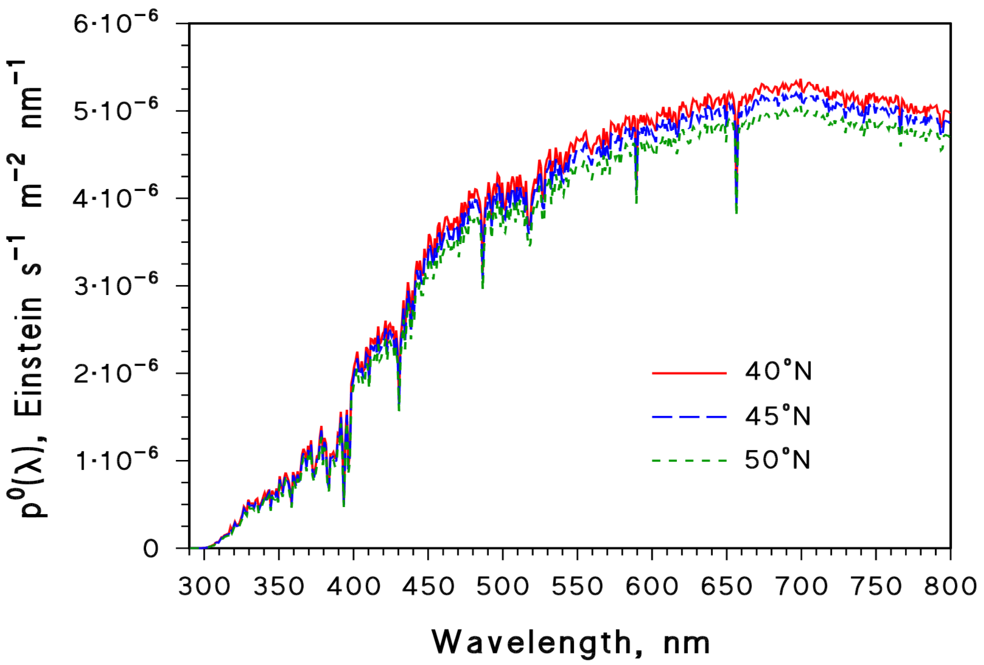

Fig. S5. Sun spectra reaching the earth surface in the middle of June at 40, 45 and 50°N. The day-averaged data were derived from the *TUV Quick Calculator* software (NCAR, 2015) for clear-sky conditions.

Table S1. European rivers and monitoring stations used for the photochemical mapping.

Nation	River	Station	Latitude	Longitude
Italy	Adige River	Badia Polesine	45.11	11.50
Italy	Adige River	Trento	46.07	11.11
Italy	Arno River	San Giovanni Alla Vena	43.68	10.50
Italy	Arno River	Subbiano	43.57	11.87
Italy	Metauro River	Ponte Degli Alberi	43.68	12.75
Italy	Po River	Boretto	44.91	10.38
Italy	Po River	Borgo Forte	45.05	10.75
Italy	Po River	Cremona	45.13	9.98
Italy	Po River	Pontelagoscuro	44.89	11.62
Italy	Tiber River	Ponte Felcino	43.28	12.33
Italy	Tiber River	Ponte Nuovo	43.00	12.40
France	Garonne River	Toulouse	43.68	1.37

France	Garonne River	Valence of Agen	44.08	0.08
France	Garonne River	Couthures	44.52	0.07
France	Loire River	Ingrandes	47.40	-0.92
France	Loire River	Orleans	47.88	1.92
France	Loire River	Roanne	46.12	4.07
France	Loire River	Saint Rambert	45.48	4.22
France	Loire River	Veauche	45.67	4.20
France	Rhone River	Collonges	46.10	5.89
France	Rhone River	Lyon	45.58	4.78
France	Rhone River	St. Vallier	45.18	4.82
France	Saone River	Auxonne	47.17	5.35
France	Saone River	Lyon	45.77	4.80
France	Seine River	Melun	48.53	2.55
France	Seine River	Mery on Seine	48.50	3.90
France	Seine River	Montereau	48.38	2.98
France	Seine River	Paris	48.85	2.35
Spain	Douro River	Puente Pino	41.56	-6.13
Spain	Douro River	Villamarcie	41.48	-4.97
Spain	Douro River	San Esteban de Gormaz	41.57	-3.22
Spain	Ebro River	Miranda de Ebro	42.68	-3.02
Spain	Ebro River	Tortosa	40.87	0.51
Spain	Ebro River	Zaragoza	41.68	0.92
Spain	Tejo River	Aranjuez	41.04	-3.60
Spain	Tejo River	Trilllo	40.69	-2.58
Portugal	Douro River	Miranda	41.48	-6.30
Portugal	Minho River	Foz do Mouro	42.10	-8.32
Portugal	Minho River	Valenca	41.90	-8.80
Portugal	Mondego River	Ponte Penacova	40.15	-8.68
Portugal	Vouga River	S.Joao de Loure	40.63	-8.48
Germany	Danube River	Jochenstein	48.52	2.98

Germany	Elbe River	Schmilka	50.89	14.23
Germany	Moselle River	Palzem	49.57	6.37
Germany	Rhine River	Maxau	49.03	8.30
United Kingdom	Carnon River	Devoran Bridge	50.18	-5.12
United Kingdom	Fal River	Tregony Gauging Station	50.27	-4.90
Switzerland	Aare River	Brugg	47.48	8.20
Switzerland	Rhine River	Basel	47.57	7.59
Switzerland	Rhine River	Diepoldsau	47.38	9.64
Switzerland	Rhine River	Rekingen	47.57	8.33
Switzerland	Rhone River	Chancy	46.16	5.98
Switzerland	Rhone River	Porte du Scex	46.35	6.89
Netherlands	Maas River	Belgian Frontier	50.77	5.68
Hungary	Danube River	Budapest	47.61	19.10

References

De Laurentiis, E., Chiron, S., Kouras-Hadef, S., Richard, C., Minella,M., Maurino, V., Minero, C., Vione, D. Photochemical Fate of Carbamazepine in Surface Freshwaters: Laboratory Measures and Modeling. *Environ. Sci. Technol*. **2012**, 46, 8164–8173. DOI: 10.1021/es3015887.

Global Environment Monitoring System. www.GEMStat.org (last access in September 2014).

QGIS Developmental Team. QGIS Geographic Information System. Open Source Geospatial Foundation Project; 2018.
